# Effects of Proximity to Abandoned Livestock Corrals on Standing Grass Biomass, Grass Species Diversity, and Wildlife Use in Olare Motorogi Conservancy, Maasai Mara, Kenya

**DOI:** 10.1002/ece3.73855

**Published:** 2026-06-23

**Authors:** Dennis Kipng'etich, Geoffrey M. Wambugu, Mwangi Kinyanjui, Caroline Ng'weno

**Affiliations:** ^1^ School of Natural Resources and Environmental Studies Karatina University Karatina Kenya; ^2^ Nature Kenya Nairobi Kenya

**Keywords:** livestock corrals, livestock–wildlife coexistence, nutrient hotspots, savanna ecosystems, standing grass biomass, wildlife habitat use

## Abstract

Pastoralists in African savanna ecosystems are shifting from livelihoods based purely on livestock only to a blended approach of livestock keeping and wildlife conservation for eco‐tourism. A popular feature of this model is the establishment of community conservancies that encourage co‐existence of traditional livestock keeping with wildlife ecotourism. Pastoralists in these ecosystems periodically establish livestock corrals which they later abandon in search of fresh pasture. The impact of these abandoned corrals on trees and shrubs is fairly well documented, but few studies exist on grasses. To complement these studies, we examined the effects of proximity to 5‐year‐old abandoned livestock corrals on standing grass biomass, grass species diversity, and wildlife use in Olare Motorogi Conservancy, Maasai Mara, Kenya over a period of 6 months. Using a distance‐based criterion, we compared close (5–55 m) and far (150–200 m) belts during wet and dry seasons. The standing grass biomass was consistently higher in close belts than far across both seasons. Biomass was significantly higher during the wet season compared to the dry season. Decreaser species showed the highest biomass close to corrals, especially during the wet season, indicating strong nutrient‐driven productivity. However, grass species richness and Shannon diversity were higher in far belts (18 species; *H*′ = 2.03) than close belts (14 species; *H*′ = 1.43), indicating the dominance of a few competing species in close belts. Wildlife dung density, used as a proxy for habitat use, was significantly higher in close belts, especially in the dry season. Biomass declined with increasing dung density (*β* = −0.0156 ± 0.0036 SE, *p* < 0.001), with stronger grazing effects close to corrals. These findings demonstrate that abandoned corrals function as ecological hotspots that enhance productivity, influence species composition, and shape wildlife distribution, highlighting their potential role in adaptive rangeland management and sustainable livestock–wildlife coexistence.

## 1 Introduction

Livestock corrals (bomas) are a defining feature of pastoral landscapes across African savannas and represent an important mechanism through which humans and livestock shape ecosystem structure and function. The long‐term presence of livestock influences the spatial distribution of plants and nutrients (Augustine 2003; Marshall et al. 2018; Muchiru et al. 2009). The pastoral mobility and settlement patterns leave persistent ecological legacies on the landscape (Veblen 2013; Vuorio et al. 2014). The periodic abandonment of traditional thorn‐fence bomas leads to the accumulation of dung, urine, and organic matter, creating localized nutrient hotspots (Marshall et al. 2018) where soil nutrient concentrations can exceed those of the surrounding savanna matrix by over 20 times (Augustine 2003; Muchiru et al. 2009; Reid and Ellis 1995). These nutrient‐enriched patches support distinct plant communities and frequently attract wild herbivores, thereby generating spatial heterogeneity in forage resources (Augustine 2003; Muchiru et al. 2009; Reid and Ellis 1995; Veblen 2013). Such heterogeneity is increasingly becoming important in contemporary savanna systems (Bartzke et al. 2026), where the need to reconcile livestock production with wildlife conservation remains a central challenge (Dickman 2010; Gao 2019). This challenge is particularly pronounced in the Maasai Mara, a globally significant ecosystem for biodiversity and tourism (Bedelian 2014; Kyalo et al. 2020), but is experiencing rapid land‐use change, increasing livestock densities, and habitat fragmentation (Homewood 2004; Lamprey and Reid 2004; Nkedianye et al. 2020; Thornton and Herrero 2010). In response, community‐based conservancies have emerged as a key management strategy to support livestock–wildlife coexistence (Veríssimo et al. 2019). Although abandoned livestock corrals are widely recognized as drivers of spatial heterogeneity, their ecological effects have primarily been studied in relation to woody vegetation. In contrast, their influence on the grass; specifically standing grass biomass and grass species diversity remains less well understood across different distances from the corrals and seasons. This represents a critical gap, given that grasses constitute the primary forage base for both livestock and wild herbivores. Although previous studies have documented changes in grass species composition around and inside corrals (Porensky and Veblen 2015), it remains unclear how these patterns vary with distance from corrals center and how proximity to the abandoned corrals influences wildlife habitat use. Therefore, this study investigates the effects of proximity to abandoned livestock corrals on standing grass biomass, grass species diversity, and wildlife use in Olare Motorogi Conservancy (OMC), Maasai Mara, Kenya.

Pastoralists have shaped savanna ecosystems for millennia through settlement patterns and livestock corral construction, creating enduring legacies in soil nutrient distribution and vegetation structure (Marshall et al. 2018; Reid and Ellis 1995).

Abandoned livestock corrals form a long‐lasting nutrient hotspot with elevated nitrogen and phosphorus levels that persist for decades to centuries, fundamentally altering primary productivity and plant community composition (Augustine 2003; Muchiru et al. 2009). In recent years, as human population pressures have increased landscape fragmentation, these localized pockets of high‐nutrient levels and vegetation have become more important especially for large herbivores (Fynn and Provenza 2023; Owen‐Smith 2004; Yoganand and Owen‐Smith 2014). These hotspots act as ecological “resource islands” within the savanna matrix, where concentrated dung and urine from livestock containment enhance soil fertility, leading to higher biomass, more productivity, and elevated forage quality compared to surrounding areas (Augustine 2003; Muchiru et al. 2009; Porensky and Veblen 2015). This pattern aligns with ecological theory on nutrient redistribution by herbivores and traditional pastoral management, creating persistent patches that structure landscape‐scale productivity and vegetation heterogeneity (Marshall et al. 2018; Veblen 2012). Functionally, abandoned livestock corrals serve as high‐quality or fecundity resources, characterized by elevated nutrient concentrations that support herbivore growth and reproduction (Fynn and Provenza 2023; Owen‐Smith 2004; Yoganand and Owen‐Smith 2014). These resources are most intensively utilized during the wet season, when herbivores can selectively exploit nutrient‐rich patches to maximize growth and reproductive investment under favorable environmental conditions. In contrast, resources that contribute mainly to biomass production are considered reserve resources, which become increasingly important during the dry season (Fynn and Provenza 2023), when overall forage quality declines across the landscape and herbivores rely on them to meet basic maintenance requirements and sustain survival.

This conceptual distinction between high‐quality (fecundity) resources and reserve (quantity) resources clarifies why corrals attract wildlife year‐round. Conservancies are therefore managed to maintain a balanced portfolio of high‐quality fecundity patches that support wet‐season productivity and reserve resources that sustain dry‐season persistence, thereby providing sufficient forage for both wildlife and livestock while preventing nutritional bottlenecks that could undermine reproductive success and long‐term population viability in fragmented landscapes (Shah et al. 2019). Within this framework, nutrient hotspots play a key role by structuring the landscape into distinct quality‐ and quantity‐based resource patches, thereby creating essential heterogeneity that underpins herbivore nutritional ecology across seasons. Notably, these nutrient legacies can persist for decades, sustaining elevated biomass and continuing to shape savanna ecosystem dynamics (Marshall et al. 2018).

Despite the well‐documented role of abandoned livestock corrals as nutrient hotspots (Augustine 2003), their ecological consequences across spatial and temporal gradients remain insufficiently resolved (Sitters et al. 2020). Existing studies demonstrate that elevated soil nutrients in abandoned corrals enhance primary productivity and create localized patches of high‐quality forage (Augustine 2003; Porensky and Veblen 2015). The nutrient enrichment in the corrals is known to influence standing biomass and plant community composition by favoring fast‐growing, nutrient‐responsive species, which may lead to competitive exclusion and reduced species diversity in highly enriched patches (Riginos et al. 2009; Van der Plas et al. 2016). Conversely, spatial gradients in nutrient availability away from corrals may support more heterogeneous plant communities with higher diversity. Forage quantity and quality are key determinants of herbivore distribution and habitat selection in savanna ecosystems (Owen‐Smith 2002). Abandoned livestock corrals enhance local productivity, alter species composition, and attract herbivores to high‐quality resource patches (Fynn and Provenza 2023; Marshall et al. 2018). Nutrient‐rich patches are likely to function as high‐quality foraging sites (Odadi et al. 2011). However, the extent to which wildlife use tracks these spatial gradients in forage resources and how this relationship varies seasonally has not been adequately quantified. These uncertainties, combined with the increasing adoption of community‐based conservancies where livestock and wildlife coexist, provide the basis for our study in OMC, Maasai Mara, Kenya. We aimed to assess: (i) how proximity to abandoned livestock corrals affects standing grass biomass across wet and dry seasons; (ii) how proximity to abandoned livestock corrals influences grass species richness and diversity; and (iii) how wildlife use varies in relation to distance from abandoned livestock corrals and season. We predicted that: (i) standing grass biomass would be higher in areas closer to abandoned livestock corrals than in areas farther away; (ii) areas near corrals would have lower grass species richness and diversity than areas farther from the corrals; and (iii) wildlife use would be greater near corrals and would vary seasonally.

## 2 Study Area, Materials and Methods

### 2.1 Study Area

**FIGURE 1:**
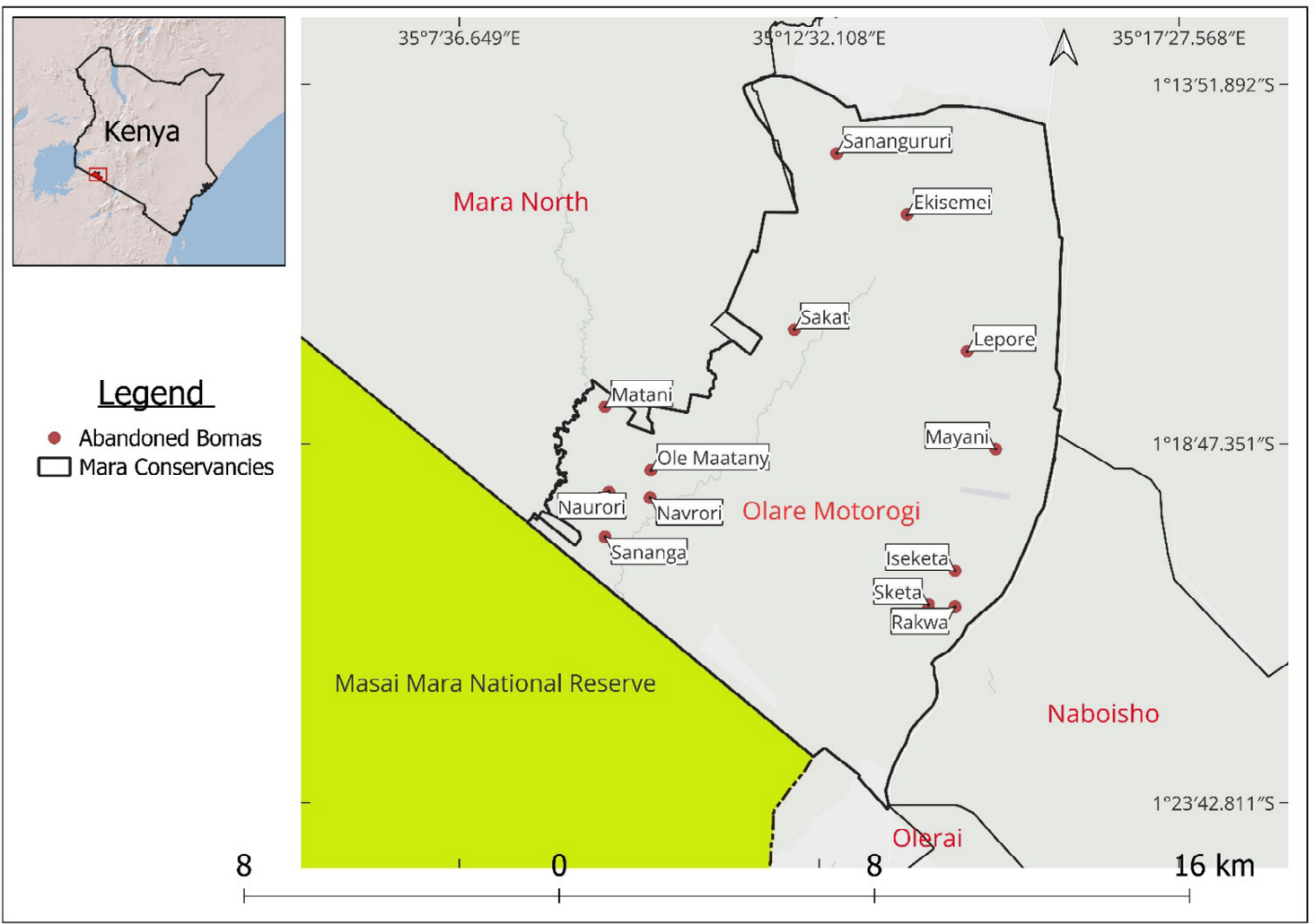
Location of Olare Motorogi Conservancy (OMC) in Greater Maasai Mara ecosystem, Kenya.

This study was conducted in the OMC (formerly Olare Orok Motorogi Conservancy), which is a community‐based conservation area in the larger Maasai Mara ecosystem. It is part of the larger Maasai Mara‐Serengeti transboundary ecosystem (Bedelian 2014; Waithaka 2004). The conservancy is next to the Maasai Mara National Reserve to the north and northwest (Figure 1). It is situated approximately (1°21′00″S, 35°12′00″E). The conservancy was established in 2006 and covers approximately 33,386 acres (Wakoli et al. 2023). Rainfall is bimodal, with the short rains in late November–December, and long rains in March–June; long‐term average annual rainfall is around 800–1000 mm (Pennycuick and Norton‐Griffiths 1976). The wettest month is typically April, while the driest months are July and August. The mean monthly maximum temperatures generally range from 25°C to 28°C, with the warmest periods occurring in January–February and October. Mean monthly minimum temperatures (often recorded at night) range from 11°C to 13°C, with the coolest nights in July–August. Daytime temperatures average around 25°C–27°C, while nights and early mornings range from 10°C to 12°C especially during the dry season (Bartzke et al. 2018; Kifworo and Dube 2024). OMC is characterized by rolling grasslands, interspersed hills, and valleys creating a diverse landscape of open savannas and elevated ridges, which was ideal for this study as it exemplifies effective community‐based wildlife conservation in a complex socio‐ecological system, and has a well‐maintained database of georeferenced abandoned livestock corrals (bomas) around OMC.

OMC is home to a wide variety of resident large mammals: African elephants ( *Loxodonta africana* ), Cape buffalo ( *Syncerus caffer* ), lions ( *Panthera leo* ) (Elliot and Gopalaswamy 2017), and leopards ( *Panthera pardus* ). It is also a habitat for spotted hyenas ( *Crocuta Crocuta* ), cheetahs ( *Acinonyx jubatus* ), and African wild dogs ( *Lycaon pictus* ). The plains of OMC hold numerous Topi (*Damaliscus lunatus jimela*), common zebras ( *Equus quagga* ), wildebeest ( *Connochaetes taurinus* ), Thomson's gazelle ( *Eudorcas thomsonii* ), Grant's gazelle ( *Nanger granti* ), hartebeest ( *Alcelaphus buselaphus* ), impala ( *Aepyceros melampus* ), common eland ( *Taurotragus oryx* ), warthog ( *Phacochoerus africanus* ), and Kirk's dik‐dik ( *Madoqua kirkii* ). The acacia woodland in OMC is browsed by Maasai giraffes ( *Giraffa tippelskirchi* ). The common hippopotamuses ( *Hippopotamus amphibius* ) are present in the OMC rivers.

In the past, abandoned livestock bomas entrenched in pastoral land usage created glades. As part of modern management, traditional pastoralists construct enclosures with thorns‐fence to confine livestock for months or years (Veblen 2012). Woody vegetation inside the boma boundaries is entirely removed, and additional trees are taken from the surrounding area to maintain the fence. When abandoned, the boma shows prolonged suppression of woody plant recruitment after nutrient‐rich livestock dung accumulations (which often reach depths of up to 0.5 m) have vanished and fencing materials have degraded.

### 2.2 Study Sites

Ground truthing allowed us to identify corrals in OMC and verified that these were abandoned livestock corrals. To ascertain the corrals' ages, we cross‐checked these corrals with the OMC database. Thirteen corrals that we analyzed were abandoned in 2016. Seven corrals were eliminated during the screening process since they were active. We visited each corral with two separate groups of 10 herders and 10 conservancy ranchers who had lived in the OMC since 2006 in order to determine the year of last use. In OMC, there are 13 known abandoned corrals that are 5 years old (5 years after abandonment) and neither utilized by people nor livestock. The 13 corrals had a diameter of about 50 m in a circle. The precise dimensions of the 13 abandoned corrals (perimeter +centers) (Table S1) are documented in the conservancy database. The abandoned corrals are dispersed throughout OMC (Figure 1). Corrals of similar age control for time since abandonment effects and minimize variability related to successional processes; this made it possible for us to separate the effects of seasonality and distance from corrals on wildlife and grass species.

### 2.3 Methodology

The study was conducted from March to August 2021 for a span of 6 months. During the study period (March–August, 2021), each of the 13 abandoned livestock corrals was visited once a month, for a total of six repeated sampling visits per corral.

A spatially explicit transect design was used across 13 corrals. A 5‐m buffer was established from the center of each corral to minimize direct disturbance effects. From the edge of this buffer, three transects were laid out at bearings of 360°, 120°, and 240° with respect to the north cardinal direction. Along each transect, measurements were collected at two distance categories: Close (5–55 m from the corral center) and far (150–200 m from the corral center).

Standing grass biomass was measured along the transects at 5 m intervals in both distance categories during each monthly visit. Grass biomass was estimated using a Disc Pasture Meter (dpm), allowing for comparison across distance (close vs. far) and season (wet vs. dry). Concurrently, grass species composition and abundance were recorded at established sampling points along the same transects and distance categories. All grass species encountered were identified and recorded, enabling calculation of species richness and Shannon diversity indices.

Wildlife habitat use was assessed using dung counts as a proxy for patterns of spatial use. At each corral, three transects were laid at bearings of 360°, 120°, and 240° relative to the north cardinal direction. Along each transect, two belt transects (50 m × 2 m) were established: one close to the corral center (5–55 m) and the other farther away (150–200 m). All wildlife dung piles and pellets within the belt transects were identified to species level and counted.

According to Porensky and Veblen (2015), abandoned livestock corrals contribute to localized nutrient enrichment by accumulating urine and dung that results in corrals. These corrals are classified as close (5–55 m) or far (150–200 m) due to their impact, which diminishes with increasing distance from the corral center. Increased standing grass biomass, intense wildlife grazing activity, and high soil fertility are all contemporary in the close distance zone (Veblen 2012). In contrast, conditions in the far distance zone serve as a benchmark for comparison (Augustine 2003). Understanding the ecological effects of abandoned corrals and their possible use in rangeland management aids in livestock‐wildlife cohabitation in OMC.

#### 2.3.1 Grass Biomass Estimation, Forage Categorization and Species Composition

To estimate standing grass biomass in abandoned corrals, three 200‐m‐long radiating transects were established at 360°, 120° and 240° from the center of each corral. At each transect, a 5‐m buffer was created from the center of the corral to avoid overlap. Two‐50 m × 2 m belts were marked at each transect, one close to the center (5–55 m) and the other far from the center of corral (150–200 m) (Figure S1).

The standing grass biomass was estimated from grass height measured at intervals of 5 m along each transect (for both close to and far from the center) using Disc Pasture Meter (dpm) (Harmse et al. 2019) dropped at arm's length. Standing biomass was then computed from the measured heights in kilograms per hectare (kg/ha) using a standardized model developed by Van Essen et al., 2000 (*Y* = −592 + 1103√*X*, where *X* = mean disc height in cm/dpm and *Y* = standing biomass in kg ha^−1^).

**FIGURE 2:**
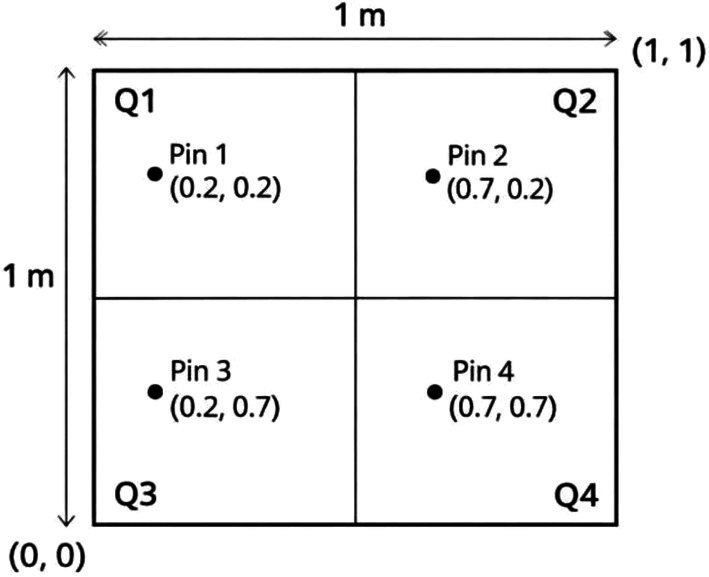
Layout of the 1 m × 1 m quadrat showing quadrant divisions (Q1–Q4) and pin sampling positions.

At each 5‐m interval, the species composition of the grass and its quality were evaluated using the point pin drop method (Figure 2). A 1 m × 1 m frame was created at each sampling location and divided into four equal quadrants (Q1–Q4). To maintain uniformity, a single pin was dropped at the set points inside each quadrant: Pin 1 at (0.2, 0.2) in Q1, Pin 2 at (0.7, 0.2) in Q2, Pin 3 at (0.2, 0.7) in Q3, and Pin 4 at (0.7, 0.7) in Q4. For each pin drop, only the first contact made with a species of grass was recorded. The forage category was characterized based on the type of grass species/object hit by the point pin dropped.

According to Trollope (1990), the grass species hit was assigned an ecological category (Decreaser, Increaser I or Increaser II) that described the state of the rangeland (Table S2). When a rangeland is in good condition, decreaser species predominate and diminish due to either overgrazing or undergrazing. Increaser II species predominate in overgrazed rangelands, while Increaser I species predominate in undergrazed or selectively utilized rangelands. Heavily used rangelands that indicate overgrazing due to the presence of Increaser II species may become dominated by more palatable Decreaser species under better management (Angassa 2012).

#### 2.3.2 Wildlife Density Estimation

All wildlife dung piles/pellets (species‐level identification) within the belt transect (two‐50 m × 2 m belts, one close to the center [5–55 m] and the other far from the center [150–200 m]) were identified and counted. Wildlife species were categorized according to their feeding guilds following Lamprey (1963). To ensure that only recent dung was counted, dung deposits were crushed during each sampling event. Since direct conversions can be difficult, wildlife dung counts were not translated into absolute densities (Fuller 1991). Instead, estimations of relative use (i.e., close vs. far) were obtained using dung counts. Dung counts are regarded as trustworthy as other surveys (Young et al. 2005). Variations in wildlife dung densities were interpreted as indicative of disparity in wildlife habitat use.

#### 2.3.3 Seasonal Classification

Seasonal variation in OMC, Maasai Mara, Kenya, was quantified using time‐series analysis of the Normalized Difference Vegetation Index (NDVI) to objectively identify wet and dry seasons during the study period (Scanlon et al. 2002). A Minimum Convex Polygon (MCP) that includes all GPS points connected to the study's abandoned livestock corrals was used to derive NDVI data for the entire OMC. We used a variance‐minimization thresholding technique implemented in R using the EBImage package to develop NDVI thresholds differentiating wet and dry conditions. To identify seasonal changes, NDVI‐based temporal segmentation was employed instead of fixed NDVI cut‐off values. In accordance with recognized methods for identifying phenological change, seasonal onsets and terminations were determined by combining the derived NDVI threshold with inflection points in the NDVI time series (Forkel et al. 2013). The Sf, Raster, stats and zoo packages in R were used for time‐series smoothing and temporal trend identification. Wet seasons were defined as times when NDVI values were regularly above the threshold (0.47), and dry seasons were defined as times when NDVI values fell below this level (Figure S2). The NDVI‐ and threshold‐based framework captured inter‐annual variability in rainfall and spatial heterogeneity in vegetation productivity characteristic of OMC. This method allowed for a thorough evaluation of the seasonal impacts on standing grass biomass, grass species diversity, and wildlife use of abandoned livestock corrals by providing ecologically significant, site‐specific seasons.

### 2.4 Data Analysis

The total standing grass biomass (kg ha^−1^) was first calculated for sampling points located close and far from the corral. Standing biomass estimates were derived using a standardized disc pasture meter (dpm) model developed by Van Essen et al. (2000) and adapted for the Mara region. The model was used to convert the mean disc height per sampling point (obtained by averaging the dpm measurements from the five surveys) into standing grass biomass.

Prior to statistical analysis, all data were first evaluated for homoscedasticity and normality. Both graphical techniques (Q–Q plots) and statistical tests (Shapiro–Wilk test; Mishra et al. 2019) were used to assess normality, Shapiro–Wilk test with *p* < 0.05 indicating deviation from normality. The Levene's test was used to evaluate homogeneity of variance; *p* < 0.05 showed uneven variances. Based on whether these presumptions were satisfied, statistical tests were selected. The total standing grass biomass at close and far locations in relation to the boma was compared using Wilcoxon rank‐sum tests. Wilcoxon rank‐sum test was also used to compare grass species abundance between close and far locations. Grass species diversity at close and far locations was evaluated using the Shannon diversity index (*H*′) (Shannon and Weaver 1949) and alpha diversity. The Shannon–Wiener diversity index is expressed as:

$$H^{\prime }=-\sum_{i=1}^{S}p_{i}\ln\left(p_{i}\right)$$

where $p_{i}=n_{i}/N$ represents the proportion of grass species *i* in the sample, $n_{i}$ is the abundance of grass species *i*, N is the total abundance of all grass species, and S is the total number of grass species.

In accordance to regional classification, grass species were further classified into ecological categories (Mureithi et al. 2014) based on their functional traits and grazing response (Trollope 1990) such as palatability and grazing tolerance categories.

Wildlife species dung counts were compiled at the corral level and compared between the wet and dry seasons. Wildlife dung density per 100 m^2^ was calculated using wildlife dung counts. Dung density was calculated for each corral by dividing the mean dung count by the total sampled area, after which values were then added together across sampling locations and then averaged across 13 corrals. Using Wilcoxon rank‐sum tests, the density of wildlife dung was assessed throughout the 13 corrals and compared between close and far locations. We also assessed the effects of season (wet vs. dry season) and distance from livestock corrals (close vs. far), and their interaction, on standing grass biomass. We used Aligned Rank Transform (ART) approach because residuals from an initial two‐way ANOVA broke the assumptions of homoscedasticity (Levene's test, *p* < 0.05) and normality (Shapiro–Wilk test, *p* < 0.05) (Wobbrock et al. 2011). By aligning and ranking the response variable (standing grass biomass) while maintaining the factorial structure, this non‐parametric method enables reliable inference for both main effects (season; distance from livestock corrals) and interactions (season × distance from corral). All analyses were conducted in R using the ARTool package (v1.1.0; Elkin et al. 2021).

The effects of distance from the corral center on standing grass biomass and wildlife dung density were investigated using generalized linear mixed‐effects models (GLMMs) with a Gamma distribution since standing grass biomass values were strictly positive, strongly right skewed and deviated from normality. To accommodate for spatial non‐independence among sampling units, corral ID was incorporated as a random effect in every model. The glmmTMB () function in R's glmmTMB package was used to fit the models (R Core Team 2021). The MuMIn package's multi‐model inference was used to choose the models. Akaike's Information Criterion (AIC) was used to rate the models (Burnham and Anderson 2002). Using the AICcmodavg package in R, model averaging was performed in accordance with Arnold's (2010) recommendations to account for model uncertainty when several competing models had ΔAIC < 2. The same analytical approach was used to evaluated the effects of season (wet vs. dry), distance from the corral (belt: close vs. far) and dung density on the standing grass biomass. To accommodate for recurrent sampling over seasons and distance belts within the same location, the corral ID was added as a random effect.

## 3 Results

### 3.1 Effects of Proximity to Livestock Corrals and Seasonality on Standing Grass Biomass, Species Richness, and Species Diversity

**FIGURE 3:**
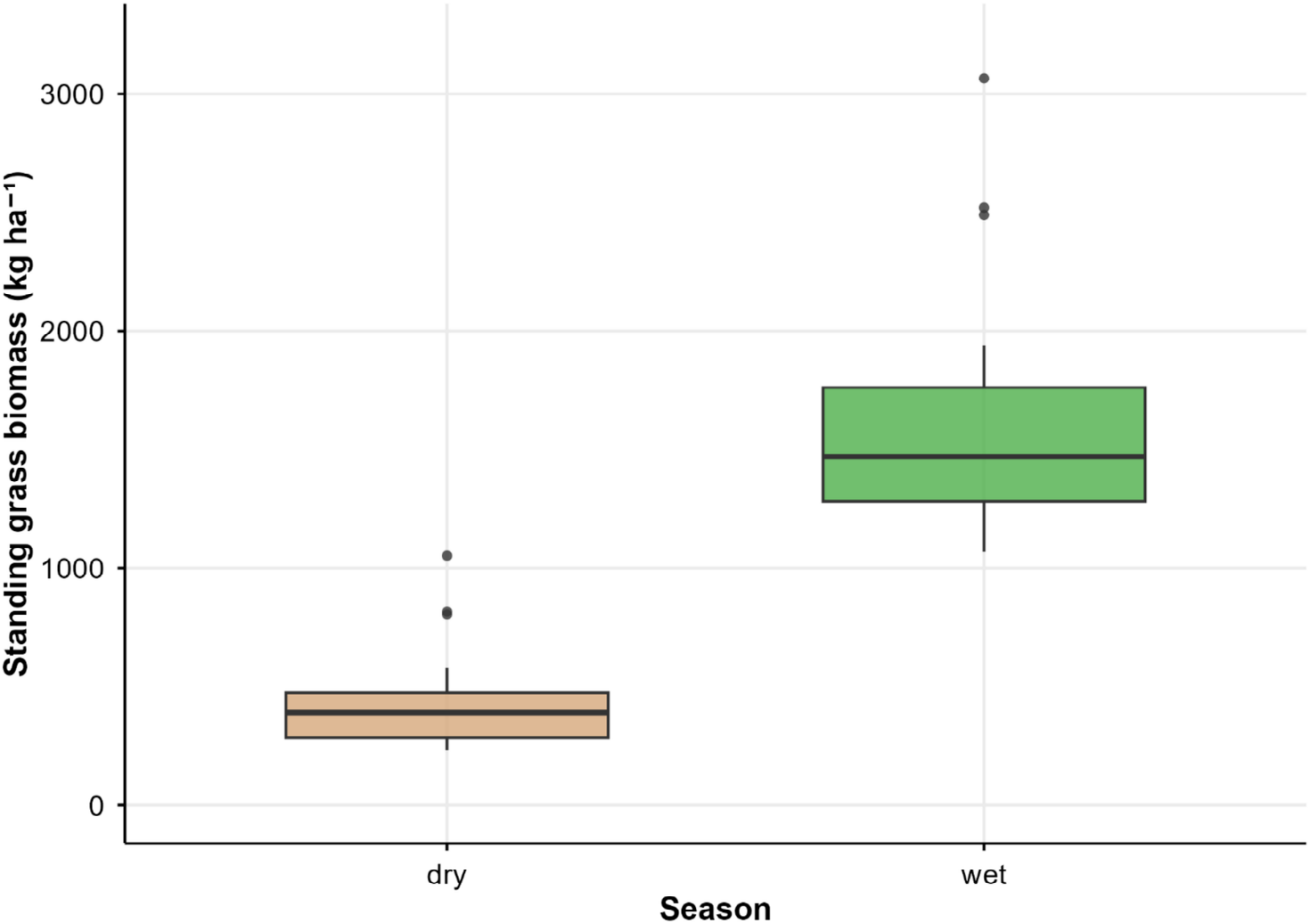
Standing grass biomass in dry and wet seasons.

**FIGURE 4:**
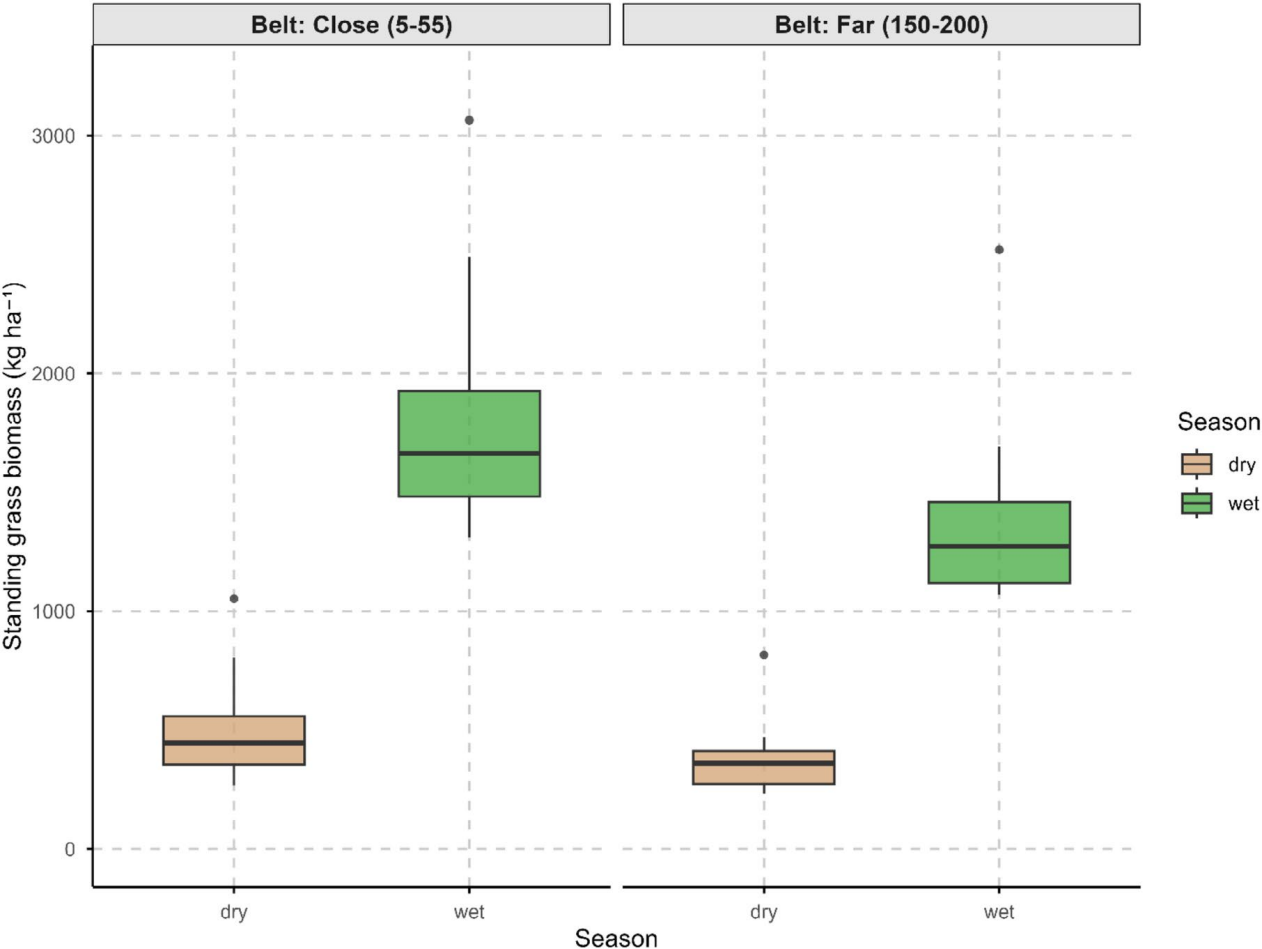
Standing grass biomass by season and belt (Close: 5–55 m; Far: 150–200 m).

Standing grass biomass increased from the dry to the wet season (Figure 3). The mean standing grass biomass during the wet season was higher in locations close to corrals (1801.43 ± 138.21 SE kg ha^−1^) than at locations far away (1398.75 ± 108.55 SE kg ha^−1^; *F*_1,48_ = 14.03, *p* < 0.001). During the dry season, standing grass biomass was much higher near corrals (501.36 ± 61.12 SE kg ha^−1^) than far away (369.94 ± 42.92 SE kg ha^−1^). Overall, the wet season's standing grass biomass (1600.09 ± 95.05 SE kg ha^−1^) was significantly higher than the dry season's (435.65 ± 38.87 SE kg ha^−1^; *F*_1,48_ = 145.23, *p* < 0.001; Figure 4). There was no statistically significant interaction between season and distance from corrals (*F*_1,48_ = 3.42, *p* = 0.071), suggesting that the effect of distance from corrals on biomass was consistent across seasons.

**FIGURE 5:**
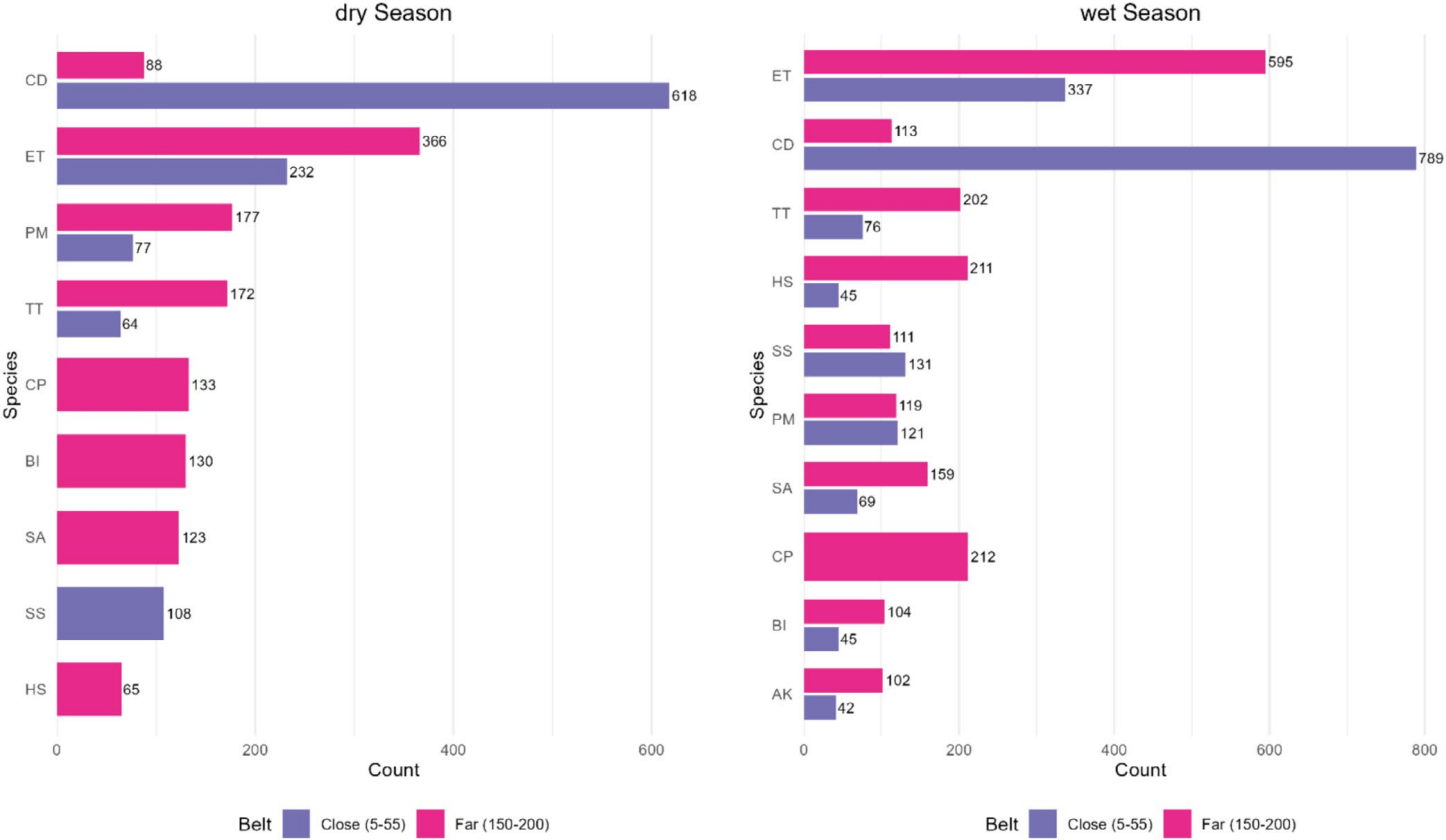
Dry and wet season abundance patterns by proximity category (Belt, close 5–55 m, far 150–200 m).

**FIGURE 6:**
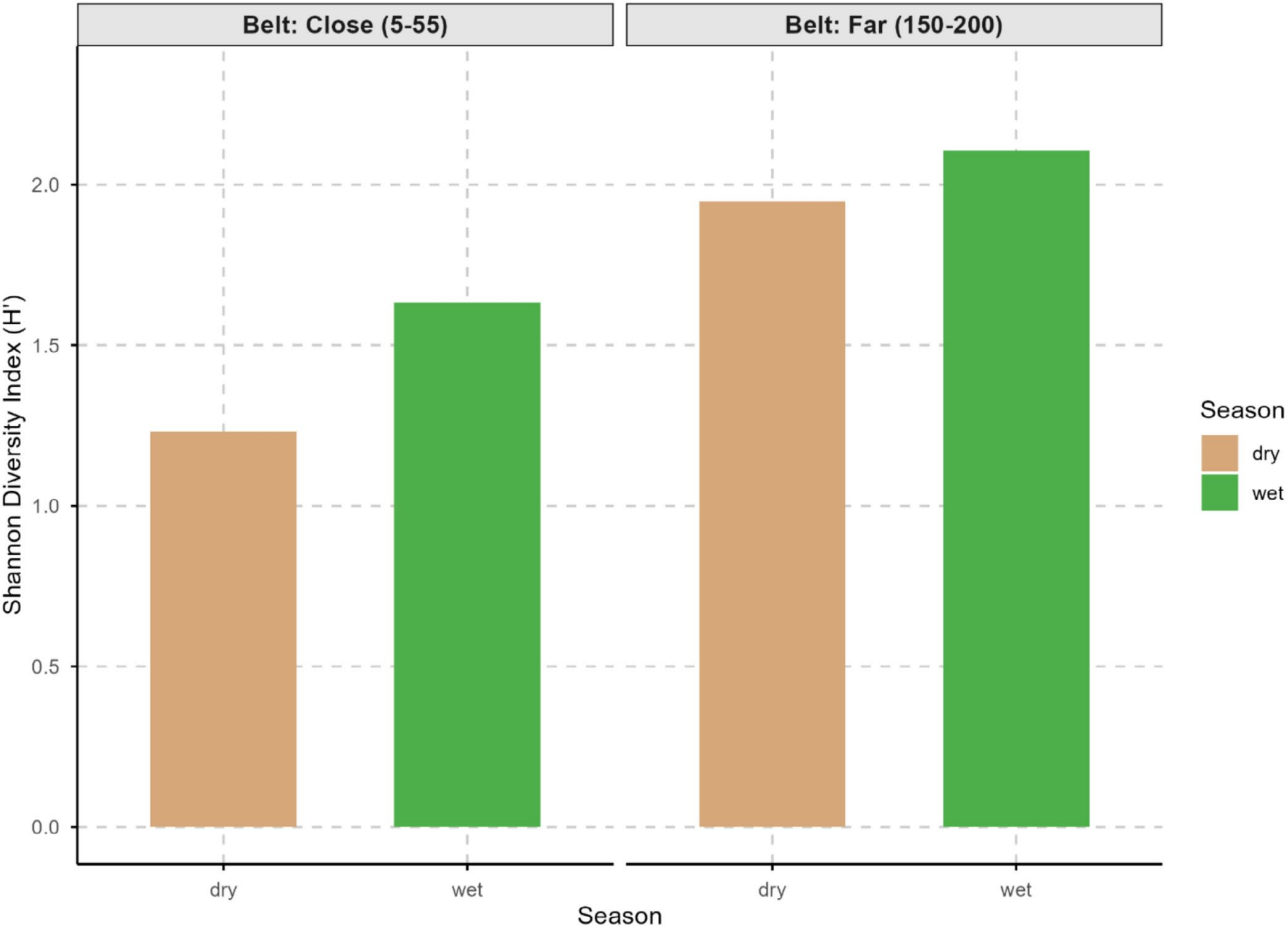
Seasonal Shannon index (*H*′) at close to (5–55 m) and far from (150–200 m) the corral.

Grass species richness and abundance increased from dry to wet season (Figure 5), with higher Shannon diversity in wet season. Wet season has a richness of 19 grass species as opposed to 13 species in dry season (Table 1). The grass species‐abundance patterns demonstrate how the wet season significantly raised the count of grasses. The wet season showed much greater counts of CD, ET, TT, CP, HS, and SS than dry season (Figure 5). Grass species (such as AK and BI) were exclusively observed during the rainy season, which added to the richness. A few species (especially CD) dominated locations close to the corrals during the dry season (Figure 5). Consequently, the lowest diversity (*H*′ = 1.23) and richness (5 species) were found in dry‐close locations. In dry‐far belt locations, grass species such as ET, TT, HS, PM, BI, CP, and SA displayed a more uniform distribution of individuals, leading to increased Shannon diversity (*H*′ = 1.95) and richness (8 species). In wet season, most species were more abundant at both close and far locations, although there were still noticeable variations in grass species structure. Wet close locations had a richness of 9 species and Shannon diversity of *H*′ = 1.63 due to the dominance of a few highly competitive species (CD and ET). Wet far locations showed the most uniform distribution of species abundance on ET, TT, CP, HS, SA, SS, PM, AK, and BI (Figure 5), thus wet far locations supported higher Shannon diversity of *H*′ = 2.11 (Figure 6) and richness of 10 species.

**TABLE 1:** Species richness and Shannon diversity index (*H*′) across seasons and distance belts from corrals.

	Dry season		Wet season	
	Close (5–55)	Far (150–200)	Close (5–55)	Far (150–200)
Species richness	5	8	9	10
Shannon diversity (*H*′)	1.23	1.95	1.63	2.11

The diversity and richness of grass species varied significantly between belts. In comparison, the locations close to the corrals (5–55 m) recorded 14 species and lower diversity (*H*′ = 1.43); locations far from corrals (150–200 m) supported higher species richness (18 species) and greater Shannon diversity (*H*′ = 2.03) (Table 1).

### 3.2 Changes in the Standing Grass Biomass Among Different Types of Grass Forage Categories

**FIGURE 7:**
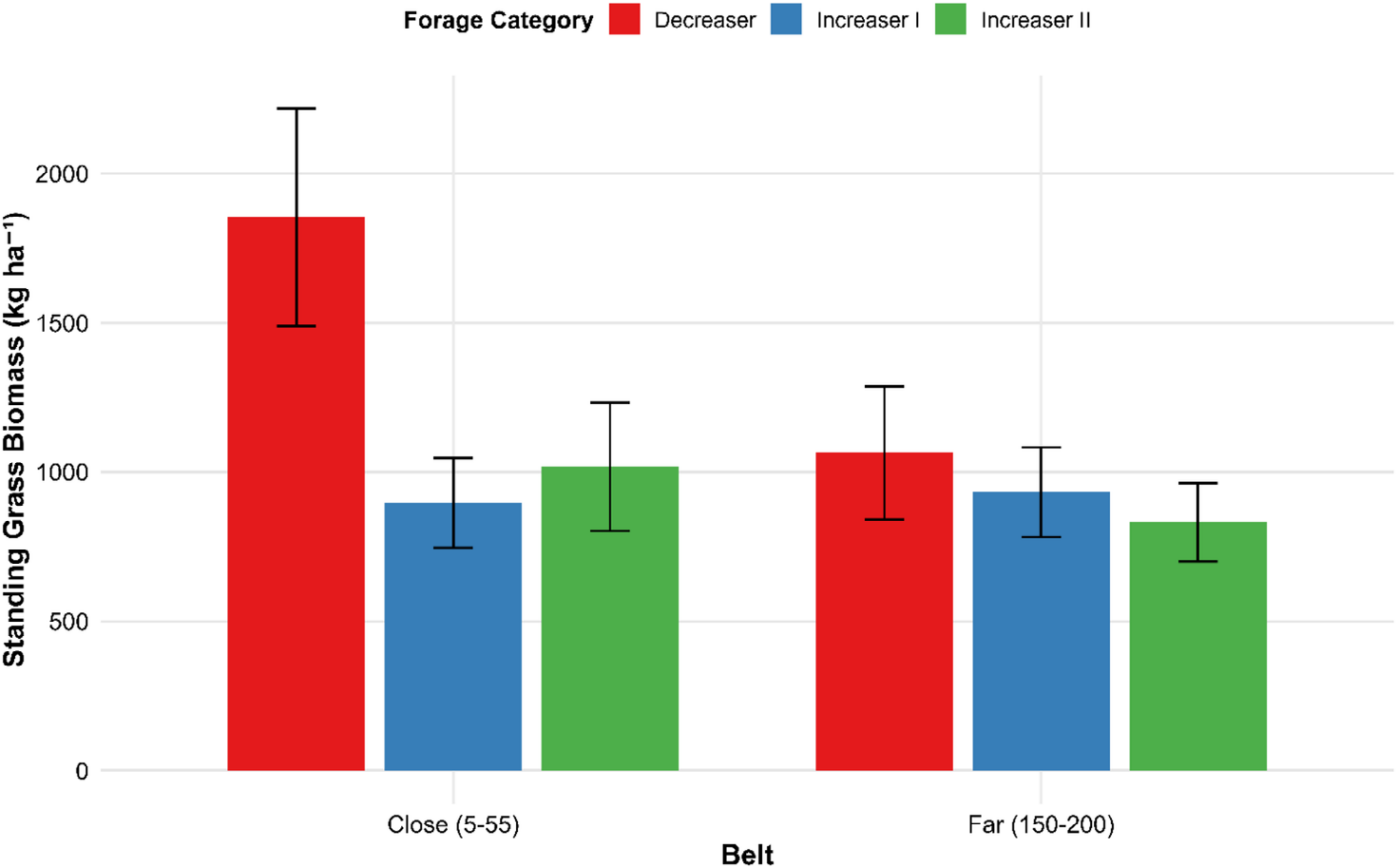
Standing grass biomass (kg ha^−1^) for forage category across distance (close vs. far).

**FIGURE 8:**
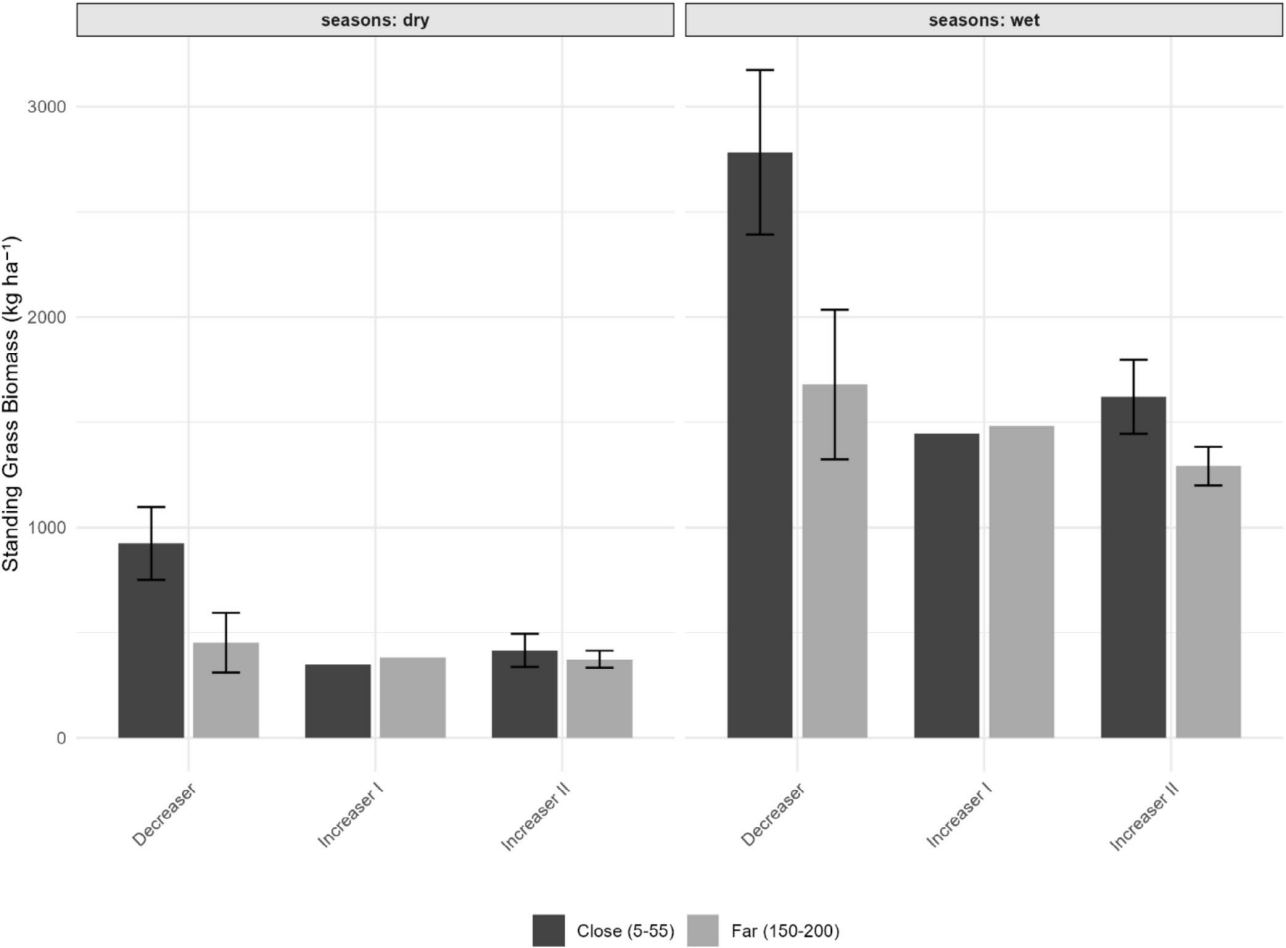
Standing grass biomass by forage group categories.

The standing grass biomass varied significantly between belts, between seasons, and among the forage types (Figures 7 and 8). Biomass differed significantly across forage categories (*F*_2,1447_ = 13.03, *p* < 0.001), between the close and far belts (*F*_1,1447_ = 12.43, *p* < 0.001), and between seasons (*F*_1,1447_ = 205.90, *p* < 0.001). The interaction between forage category and belt was significant (*F*_2,1447_ = 4.30, *p* = 0.014).

Increaser I and Increaser II species exhibited lower biomass values, with differing responses to distance from corrals, whereas Decreaser species consistently showed the highest biomass (Figure 8). The close belt had a much higher biomass of decreaser grasses (1853.04 ± 364.23 SE kg ha^−1^) than the far belt (1065.18 ± 223.31 SE kg ha^−1^). In contrast, the grass biomass of Increaser I species was (897.46 ± 150.30) in the close belt and (932.45 ± 151.05 SE kg ha^−1^) in the far belt, indicating comparable biomass levels in both belts. The grass biomass for Increaser II species was slightly higher in the close belt (1018.29 ± 214.78 SE kg ha^−1^) than in the far belt (832.21 ± 130.05 SE kg ha^−1^) (Figure 7).

#### 3.2.1 Seasonal Variations in Standing Grass Biomass Among Grass Forage Categories

All forage categories and distance belts showed significant seasonal patterns in standing grass biomass, with consistently higher biomass during the wet season compared to the dry season (Figure 8). In both belts, biomass for Decreaser species increased significantly between the dry and wet seasons (Figure 8). The mean biomass in the close belt increased from 810.15 ± 74.04 SE kg ha^−1^ in the dry season to 2498.78 ± 169.12 SE kg ha^−1^ in the wet season (estimate = −623, *t*_1447_ = −9.25, *p* < 0.0001). The far belt showed a similar trend, with biomass rising from 499.61 ± 46.86 SE kg ha^−1^ in the dry season to 1802.28 ± 106.17 SE kg ha^−1^ in the wet season (estimate = −659, *t*_1447_ = −16.11, *p* < 0.0001). Increaser I species showed strong seasonal responses, with wet‐season biomass in both belts surpassing dry‐season values. Biomass increased from 347.16 ± 159.22 SE kg ha^−1^ in the dry season to 1447.77 ± 181.01 SE kg ha^−1^ in the wet season in the close belt (estimate = −725, *t*_1447_ = −3.44, *p* = 0.0006), and from 381.40 ± 75.44 SE kg ha^−1^ to 1483.50 ± 204.99 SE kg ha^−1^ in the far belt (estimate = −669, *t*_1447_ = −4.09, *p* < 0.0001). Biomass for Increaser II species exhibited a similar seasonal pattern, with noticeably higher values during the wet season. The mean biomass increased from 498.77 ± 27.99 SE kg ha^−1^ in the dry season to 1789.71 ± 140.58 SE kg ha^−1^ in the wet season in the close belt (estimate = −655, *t*_1447_ = −35.07, *p* < 0.0001) and from 342.86 ± 16.63 SE kg ha^−1^ in the dry season to 1321.19 ± 139.53 SE kg ha^−1^ in the wet season for the far belt (estimate = −689, *t*_1447_ = −28.49, *p* < 0.0001).

#### 3.2.2 Species‐Level Seasonal Variation in Standing Grass Biomass Across Forage Categories

**FIGURE 9:**
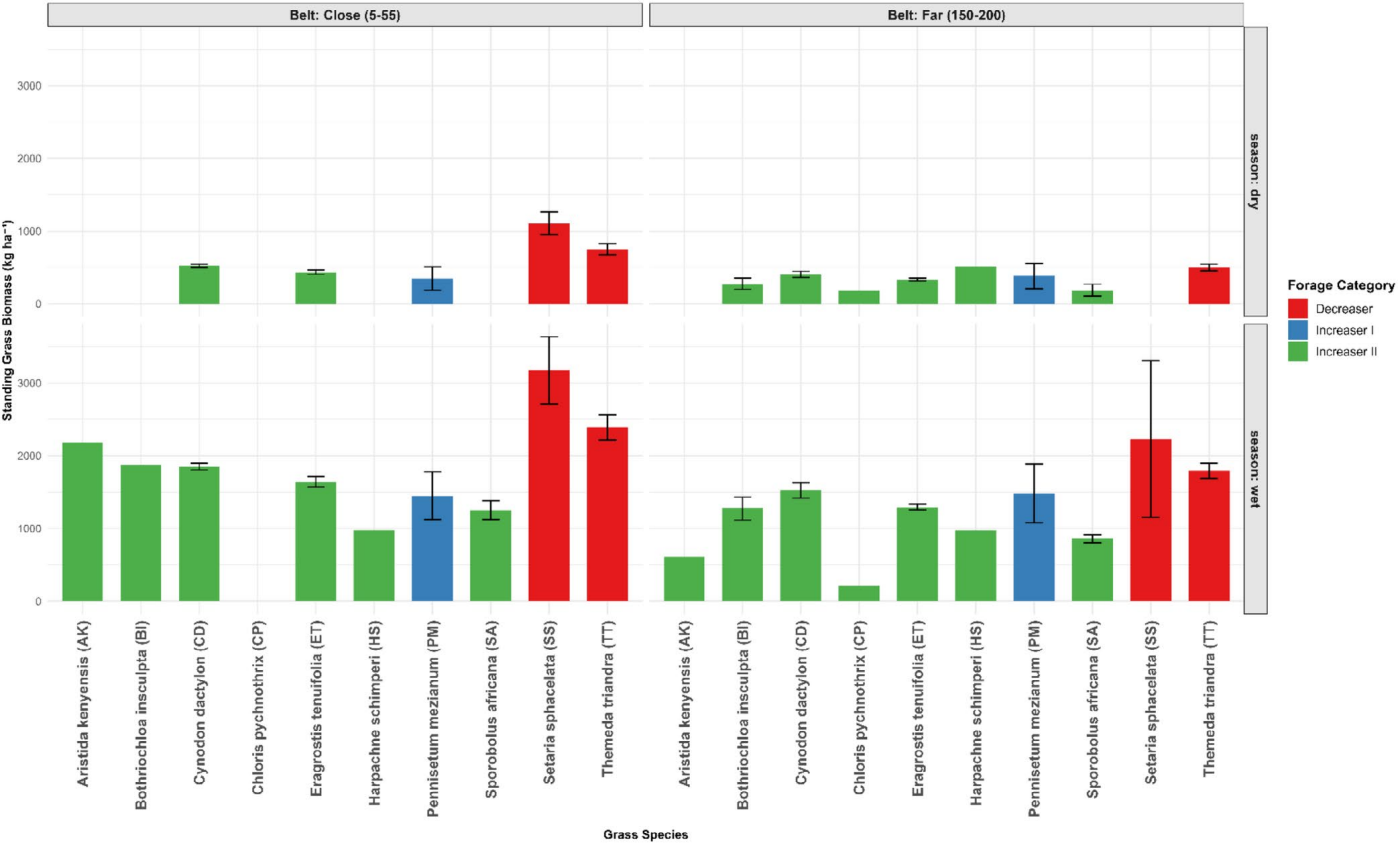
Standing grass biomass (kg ha^−1^) of grass species in Close (5–55 m) and Far (150–200 m) belts during dry and wet seasons.

Species‐level patterns show clear seasonal, belt, and forage variations in standing grass biomass. Wet‐season biomass was consistently higher than dry‐season biomass (Figure 9). In the decreaser category both species (SS and TT) showed a significant seasonal increase in biomass. SS increased from 1108.04 ± 218.35 during the dry season to 3173.71 ± 417.70 during the wet season (estimate = −606, *t*_1427_ = −3.468, *p* = 0.0005), and TT increased from 750.58 ± 193.86 to 2390.80 ± 379.95 (estimate = −642, *t*_1427_ = −8.710, *p* < 0.0001) for close belt. The far belt showed a similar seasonal pattern, with TT rising from 499.61 ± 116.51 (dry) to 1791.45 ± 149.63 (wet) (estimate = −641, *t*_1427_ = −15.466, *p* < 0.0001) and SS reaching 2229.85 ± 314.30 during the wet season.

In the Increaser I category (represented by PM), biomass in the close belt increased from 347.16 ± 59.22 during the dry season to 1447.77 ± 181.01 during the wet season (estimate = −698, *t*_1427_ = −3.282, *p* = 0.0011). A similar increase from 381.40 ± 75.44 (dry) to 1483.50 ± 204.99 (wet) (estimate = −662, *t*_1427_ = −4.021, *p* < 0.0001) was noted in the far belt.

In the Increaser II species; in both belts, dominant species like CD and ET showed significant seasonal increases. In the close belt, ET increased from 436.22 ± 103.50 to 1642.77 ± 177.86 (estimate = −666, *t*_1427_ = −18.671, *p* < 0.0001), and CD increased from 523.11 ± 157.14 (dry) to 1851.74 ± 208.38 (wet) (estimate = −658, *t*_1427_ = −29.593, *p* < 0.0001). Similar patterns were seen in the far belt, where CD increased from 404.63 ± 90.23 (dry) to 1523.70 ± 172.75 (wet) (estimate = −666, *t*_1427_ = −11.857, *p* < 0.0001), and ET increased from 333.58 ± 64.13 to 1294.13 ± 166.94 (estimate = −690, *t*_1427_ = −24.702, *p* < 0.0001). Less abundant species (such as AK, BI, HS, CP, and SA) typically showed lower biomass in the dry season and higher biomass during the wet season (BI: estimate = −715, *t*_1427_ = −4.341, *p* < 0.0001; SA: estimate = −717, *t*_1427_ = −3.768, *p* = 0.0002) (Figure 9).

### 3.3 Seasonality and Corral Distance Effects on Wildlife Use and Biomass

**FIGURE 10:**
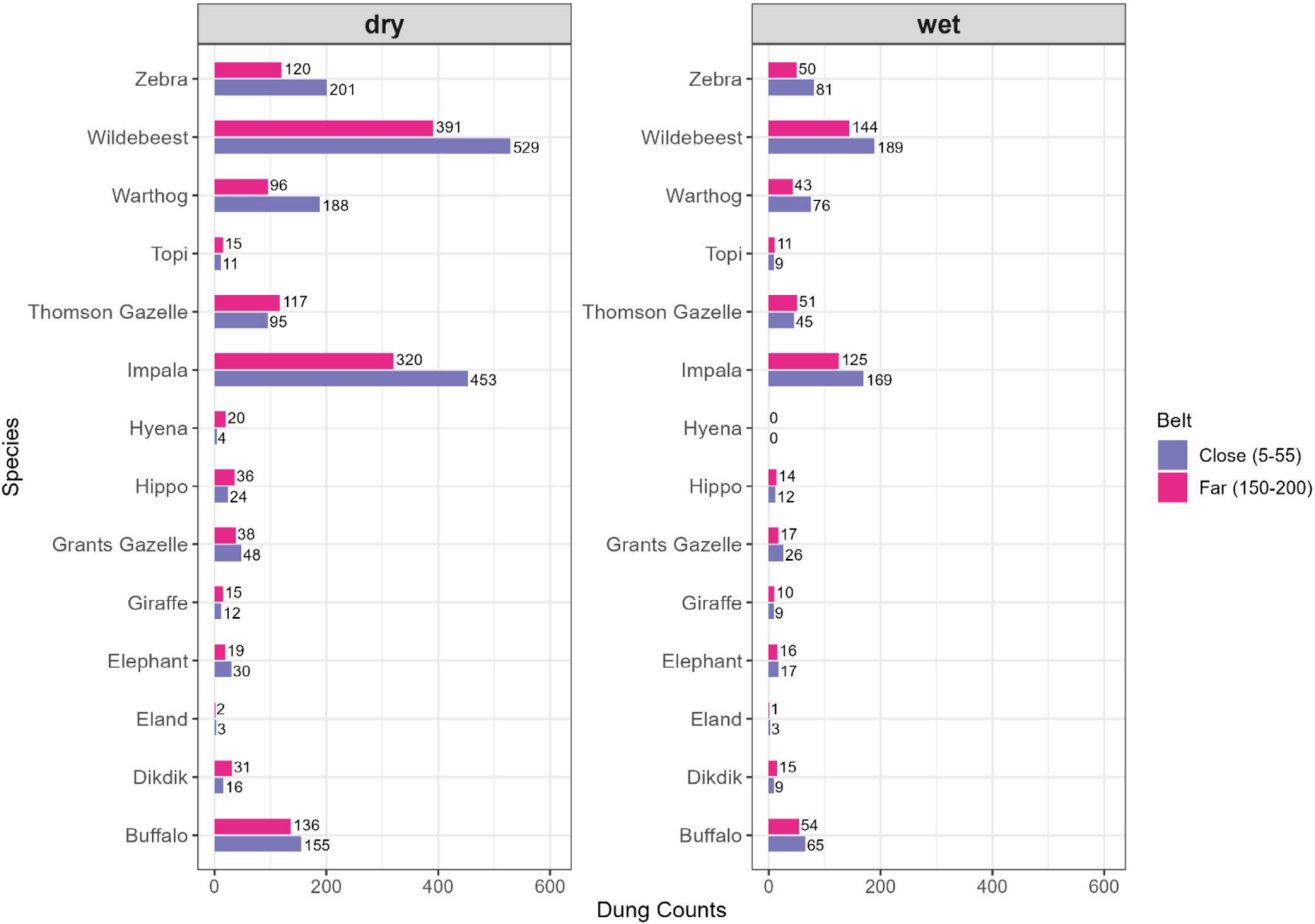
Dung counts of wildlife species in dry and wet seasons across close (5–55 m) and far (150–200 m) distance belts from corrals.

A total of 14 wildlife species were recorded in dry season. In contrast 13 species were recorded during wet season as hyena was neither detected in close belt nor far belt (Figure 10). Overall wildlife dominated during dry season, which was significantly higher than the wet season (*W* = 207,895, *p* < 0.001). During the dry season, close belt (5–55 m) had much higher total wildlife abundance than those in far belt (150–200 m) (*W* = 165,993, *p* = 0.018). In wet season, close belt continued to support more species than those in the far belt (*W* = 85,992, *p* = 0.044).

**FIGURE 11:**
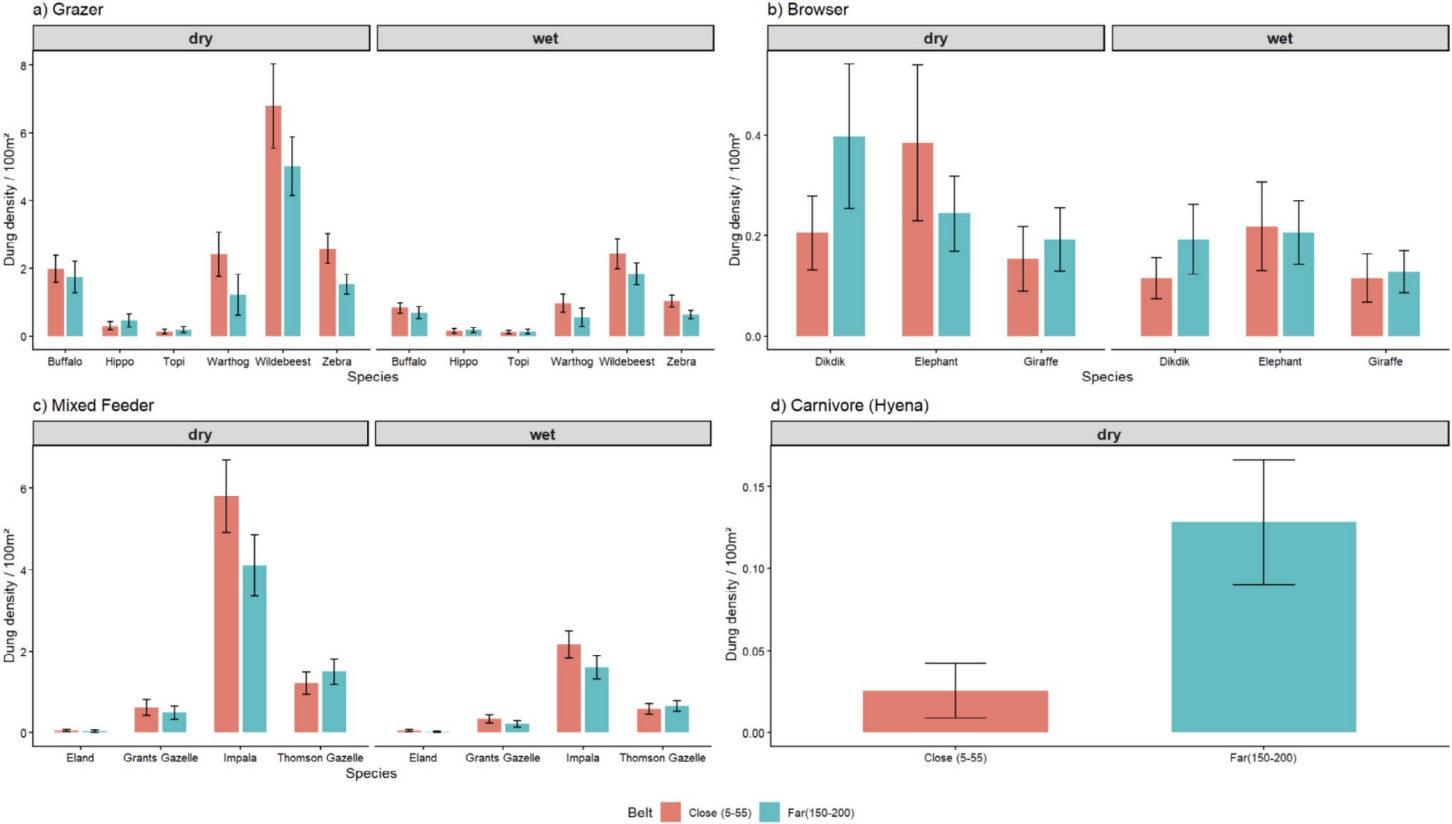
Dung density of wildlife species grouped by feeding guild at close belt (5–55 m) versus far belt (150–200 m) during dry and wet seasons.

Dung density varied significantly by feeding guild (*F*_2,1094_ = 60.85, *p* < 0.001), belt (*F*_1,1094_ = 4.65, *p* = 0.031), and season (*F*_1,1094_ = 113.49, *p* < 0.001). A significant guild × season interaction (*p* < 0.001) indicated that seasonal changes in dung density differed among feeding guilds (Figure 11). However, the guild × belt interaction was significant (*p* = 0.0203); only mixed feeders did not differ significantly (*F*_1,1094_ = 3.71, *p* = 0.083).

AIC‐based model selection identified two top‐ranked models with substantial support (ΔAIC < 2; Table 2). The best‐supported model included dung density, season, and belt distance from livestock corrals as fixed effects, with the effect of dung density allowed to vary among corral site locations (dung density effects varying by Belt) (AIC = 771.3, weight = 0.77; Table 2). The second‐ranked model (AIC = 773.9, ΔAIC = 1.68, weight = 0.20; Table 2) had the same variables but dung's density effect allowed to vary with season; these two models together accounted for 97% of the total model weight, while all other candidate models exhibited significantly less support (ΔAIC > 7).

**TABLE 2:** Top GGLMMs for standing grass biomass based on AICc weights, the top two models together explain 97% of the total model weight.

Fixed effects	*K*	logLik	AICc	Delta AIC	Cum. weight
Dung density + Season + Dung density: Belt	6	−378.70	771.26	0.00	0.77
Dung density × Season + Dung density: Belt	7	−378.70	773.94	1.68	0.20
Dung density × Season + Dung density: Belt	9	−378.01	778.30	7.04	0.02
Dung density × Season × Belt	10	−378.39	782.15	10.89	0.01
Dung density × Season + Belt	12	−377.51	787.02	15.76	0.00
Dung density × Belt	6	−398.35	810.57	39.31	0.00
Dung density + Season + Belt	6	−399.06	811.98	40.71	0.00
Dung density × Belt	8	−398.35	816.05	44.79	0.00
Dung density × Season	6	−419.02	851.92	80.65	0.00
Belt	4	−424.54	857.94	86.68	0.00
Dung density	4	−424.78	858.42	87.15	0.00
Season	4	−426.69	862.22	90.96	0.00

**FIGURE 12:**
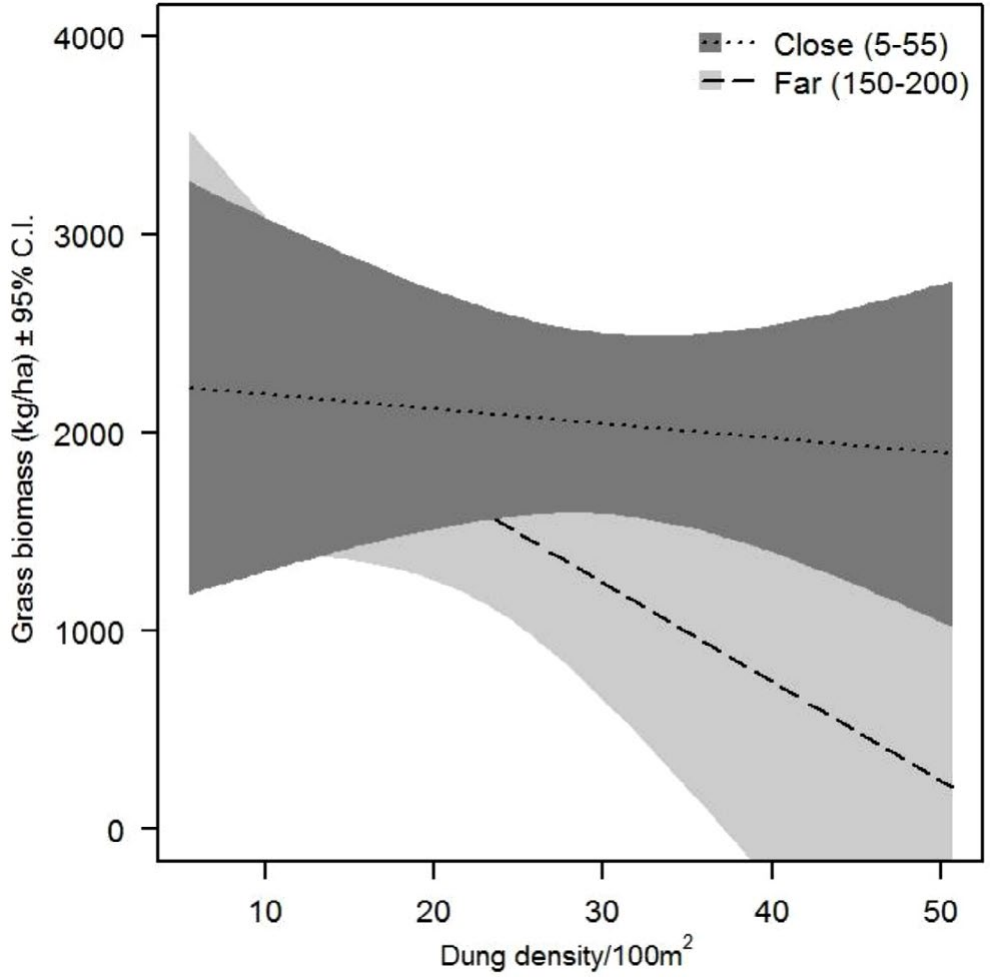
Variation in standing grass biomass compared to variation in wildlife dung density close to and far from corral.

**FIGURE 13:**
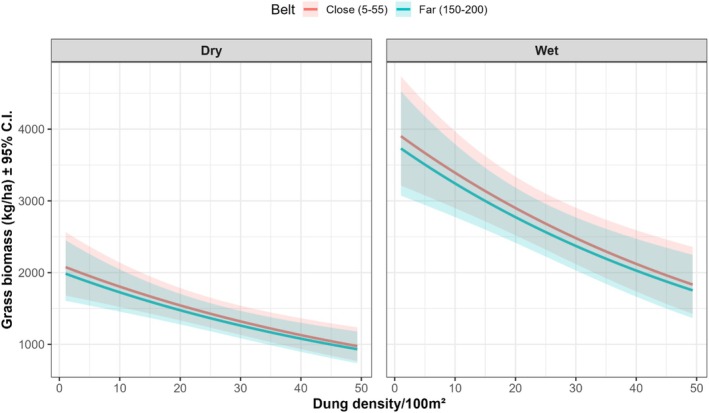
Relationships between dung density and standing grass biomass (kg ha^−1^) in the dry and wet seasons across close (5–55 m) and far (150–200 m) distance belts from livestock corrals.

The standing grass biomass decreased significantly with increasing dung density (*β* = −0.0156 ± 0.0036 SE, *Z* = −4.32, *p* < 0.001; Table 3), which corresponds to a 1.5% decrease in grass biomass for every unit increase in dung density (exp (−0.0156) = 0.985; Figure 12). The wet season had significantly higher biomass than the dry season (*β* = 0.6310 ± 0.0790 SE, *Z* = 7.98, *p* < 0.001; Figure 13), indicating that wet‐season biomass was 1.88 times higher than dry‐season biomass (exp (0.6310) = 1.88). We found a significant interaction between belts and dung density (*β* = −0.0449 ± 0.0029 SE, *Z* = −15.54, *p* < 0.001), suggesting that the effect of dung density varied by belt.

**TABLE 3:** Fixed effects estimate from the Gamma generalized linear mixed models (GGLMMs) examining the effects of dung density, season, and belt distance on standing grass biomass.

Variable	Fixed effect	Estimate	S E	*Z* value	*p*
Standing grass Biomass	(Intercept)	7.6546	0.1105	69.278	0.00
	Dung density	−0.0156	0.0036	−4.323	1.54e‐05
	Season (Wet)	0.6310	0.0790	7.984	1.42e‐15
	Dung density: Belt Far (150–200 m)	−0.0449	0.0029	−15.544	1.75e‐54

## 4 Discussion

This study demonstrates that abandoned livestock corrals function as long‐term nutrient enrichment zones that significantly enhance standing grass biomass in OMC. In line with our first hypothesis, standing grass biomass was markedly higher in areas close to corrals (5–55 m) compared to far areas (150–200 m) in both wet and dry seasons. This pattern is primarily driven by the accumulation of livestock dung and urine, which enriches the soil with organic matter, nitrogen, and phosphorus (Augustine 2003; Veblen and Young 2010; Veblen 2012). Seasonality strongly influenced overall biomass, with significantly higher values recorded during the wet season across all belts. However, the positive effect of proximity to corrals on biomass remained consistent regardless of season, indicating that nutrient legacies from livestock corrals help buffer grass productivity against seasonal variability, maintaining relatively high biomass even under dry conditions. Notably, high grazing pressure near corrals did not result in reduced biomass, suggesting that herbivory is compensated by increased growth in these nutrient‐rich patches. This pattern is consistent with compensatory growth dynamics, where defoliation stimulates rapid regrowth under high nutrient availability (Augustine and McNaughton 1998; Porensky and Veblen 2015).

Grass species richness and Shannon diversity were lower near corrals than farther away in both seasons (dry season: 5 vs. 8 species, *H*′ = 1.23 vs. 1.95; wet season: 9 vs. 10 species, *H*′ = 1.63 vs. 2.11), supporting our second hypothesis. We found that fast‐growing, nutrient‐responsive grass species such as *Cynodon dactylon* and *Eragrostis tenuifolia* dominated near the corral due to their competitive advantage in nutrient‐enriched and disturbed environments (Porensky and Veblen 2015). In contrast, areas farther from corrals supported higher grass richness and grass species diversity, reflecting more even species distribution under lower nutrient availability. These findings indicates that nutrient enrichment promotes productivity but may reduce community evenness, consistent with unimodal productivity–diversity relationship observed in grasslands (Fraser et al. 2015). Nutrient hotspots created by abandoned livestock corrals support highly productivity at the expense of grass community evenness in savanna ecosystems (Augustine 2003; Muchiru et al. 2009). Similar patterns have been reported in other savanna ecosystems (Porensky and Veblen 2015; Veblen 2012). However, the effects of increased productivity on grass diversity and evenness are often strongly mediated by grazing (Milchunas et al. 1988; Olff and Ritchie 1998). Classic theory shows that herbivores tend to decrease species richness at high productivity areas but increase it at low productivity by suppressing competitive dominants (Bakker et al. 2006). In African savannas, medium and smaller herbivores often exert strong top‐down control, especially by limiting forb abundance (Burkepile et al. 2017; Eby et al. 2014; Koerner et al. 2014). Our results suggest that abandoned livestock corrals act as persistent ecological hotspots that enhance forage availability while shifting community structure toward more productive but lower diverse grass assemblages (Muchiru et al. 2009).

Wildlife habitat use was significantly influenced by distance from livestock corrals and season, supporting our prediction. Overall wildlife use was higher close to corrals (5–55 m), particularly during the dry season, when nutrient‐enriched patches become attractive foraging areas when forage is scarce. Grazers such as zebra, buffaloes, and wildebeest showed a strong preference for areas near corrals (Veblen 2012), likely due to the higher biomass and nutritional quality of the dominant grass species in these zones. This pattern is consistent with optimal foraging theory and previous findings in African savannas (Veblen 2012; Ng'weno, Ford, et al. 2019). Browsers and mixed feeders displayed more variable responses, while some browsers such as elephants utilized corral‐proximate areas (Veblen 2012); many recorded higher densities farther from corrals, where woody vegetation was more abundant and grazing competition was lower. This spatial partitioning by feeding guilds likely reduces interspecific competition and facilitates coexistence. Carnivore activity, especially hyenas, was higher in the far belt (150–200 m). This slightly deviates from some earlier studies which reported that carnivores are attracted to corral areas due to prey concentration (Ng'weno, Buskirk, et al. 2019). The observed pattern may be explained by hyenas' scavenging behavior and preference for hunting or locating prey remains at a far distance from the corral center where prey visibility and accessibility are higher. Seasonality played a key role in wildlife distribution. During the dry season, herbivore densities increased significantly near corrals, highlighting the importance of these nutrient hotspots as dry‐season refugia. However, a negative correlation between dung density and standing grass biomass in the close belt suggests that intense herbivore use partially offsets nutrient‐driven productivity gains through grazing and trampling.

In conclusion, abandoned livestock corrals represent an important management‐relevant ecological feature in savanna ecosystems. They improve standing biomass production, influence wildlife distribution, and contribute to the spatial heterogeneity that supports ecosystem functioning by acting as nutrient‐enriched patches. Corrals can enhance biomass availability, aid in the redistribution of nutrients throughout the landscape, and support rangeland rehabilitation through strategic placement and rotational use. In order to balance productivity with biodiversity protection, strategies that incorporate geographic rotation of corral sites, control of grazing intensity, and maintenance of several nutrient hotspots are probably crucial (Reid 2012). Recognizing livestock corrals as active drivers of ecological processes, rather than incidental by‐products of pastoralism, provides a basis for integrating traditional practices into contemporary rangeland management. Such integration offers a pathway toward sustaining livestock production while supporting wildlife conservation in the Maasai Mara and similar savanna ecosystems.

## Author Contributions

**Caroline Ng'weno:** conceptualization (equal), data curation (equal), formal analysis (equal), methodology (equal), resources (equal), writing – review and editing (equal). **Mwangi Kinyanjui:** conceptualization (equal), data curation (equal), formal analysis (equal), funding acquisition (lead), methodology (equal), software (equal), supervision (equal), writing – review and editing (equal). **Geoffrey M. Wambugu:** conceptualization (equal), formal analysis (equal), methodology (equal), supervision (lead), writing – review and editing (equal). **Dennis Kipng'etich:** conceptualization (equal), data curation (lead), formal analysis (lead), methodology (equal), writing – original draft (lead), writing – review and editing (equal).

## Funding

This work was supported by Centre for Mountain and Climate change, Grant.

## Conflicts of Interest

The authors declare no conflicts of interest.

## Supporting information


**Table S1:** GPS coordinates and approximate sizes of livestock corrals (bomas) in Olare Motorogi Conservancy.
**Table S2:** Grass species categorization and their ecological categories.
**Figure S1:** Radial transect layout showing a 5 m buffer zone from the edge of the corral, with sampling in the Close (5–55 m) and Far (150–200 m) zones. Transects were established along three directions, each consisting of a Two‐50 m × 2 m belt transect, with a 5 m buffer zone maintained in each corral.
**Figure S2:** Monthly mean Normalized Difference Vegetation Index (NDVI) for the OMC in 2021, with a 0.48 threshold line (dashed red line) to distinguish wet seasons (NDVI > 0.48) from dry‐season conditions (NDVI < 0.48).

## Data Availability

The data have been included on a repository provided with an open‐access link: https://doi.org/10.5061/dryad.08kprr5j6.
