# Supplementary material for: Effects of Proximity to Abandoned Livestock Corrals on Standing Grass Biomass, Grass Species Diversity, and Wildlife Use in Olare Motorogi Conservancy, Maasai Mara, Kenya
Source: Ecol Evol. 2026 Jun 23;16(6):e73855. doi: 10.1002/ece3.73855 (PMC13290650; doi:10.1002/ece3.73855)

**Supporting information**

**Table S1**: GPS coordinates and approximate sizes of livestock corrals (bomas) in Olare Motorogi Conservancy

| Corral Location Name | Corral ID | Year of abandonment | GPS X | GPS Y | Approximate Diameter (M) | Approximate perimeter (M) |
| --- | --- | --- | --- | --- | --- | --- |
| Ekisemei | OMC1 | 2016 | 35.22897 | -1.26078 | 50.9 | 159.9 |
| Iseketa | OMC 2 | 2016 | 35.2399 | -1.34215 | 49.8 | 156.5 |
| Lepore | OMC3 | 2016 | 35.3989 | -1.34216 | 50.8 | 159.6 |
| Mayani | OMC 4 | 2016 | 35.24899 | -1.31683 | 49.9 | 156.8 |
| Naurori | OMC 5 | 2016 | 35.17002 | -1.32501 | 50.2 | 157.7 |
| Navrori | OMC 6 | 2016 | 35.17035 | -1.32549 | 50.1 | 157.4 |
| Ole Maatany | OMC 7 | 2016 | 35.17052 | -1.31921 | 49.7 | 156.2 |
| Rakwa | OMC 8 | 2016 | 35.2399 | -1.35041 | 50.5 | 158.7 |
| Sakat | OMC 9 | 2016 | 35.20422 | -1.28673 | 49.8 | 156.5 |
| Sananga | OMC 10 | 2016 | 35.16701 | -1.32892 | 49.3 | 154.9 |
| Sanangururi | OMC 11 | 2016 | 35.22897 | -1.2608 | 49.4 | 155.2 |
| Sketa | OMC 12 | 2016 | 35.23983 | -1.3504 | 49.8 | 156.5 |
| Matani | OMC 13 | 2016 | 35.159 | -1.28908 | 50.1 | 157.4 |

**Table S2:** Grass species categorization and their ecological categories

| **CODE** | **GRASS SPECIES** | **ECOLOGICAL CATEGORY** |
| --- | --- | --- |
| **AK** | *Aristida kenyensis* | Increaser II |
| **BI** | *Bothriochloa insculpta* | Increaser II |
| **CD** | *Cynodon dactylon* | Increaser II |
| **CP** | *Chloris pychnothrix* | Increaser II |
| **ET** | *Eragrostis tenuifolia* | Increaser II |
| **HS** | *Harpachne schimperi* | Increaser II |
| **PM** | *Pennisetum mezianum* | Increaser I |
| **SA** | *Sporobolus africana* | Increaser II |
| **SS** | *Setaria sphacelata* | Decreaser |
| **TT** | *Themeda triandra* | Decreaser |

**Figure S1**: Radial transect layout showing a 5 m buffer zone from the edge of the corral, with sampling in the Close (5–55 m) and Far (150–200 m) zones. Transects were established along three directions, each consisting of a Two-50 m × 2 m belt transect, with a 5 m buffer zone maintained in each corral.


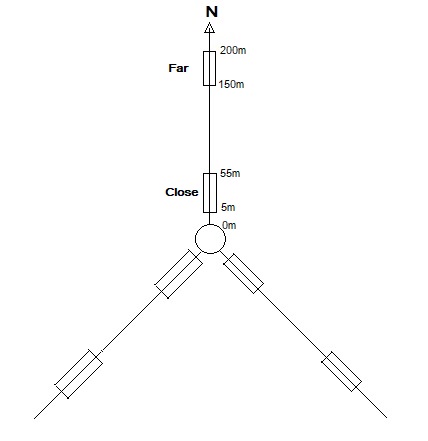


**Figure S2:** Monthly mean Normalized Difference Vegetation Index (NDVI) for the OMC in 2021, with a 0.48 threshold line (dashed red line) to distinguish wet seasons (NDVI > 0.48) from dry-season conditions (NDVI < 0.48).


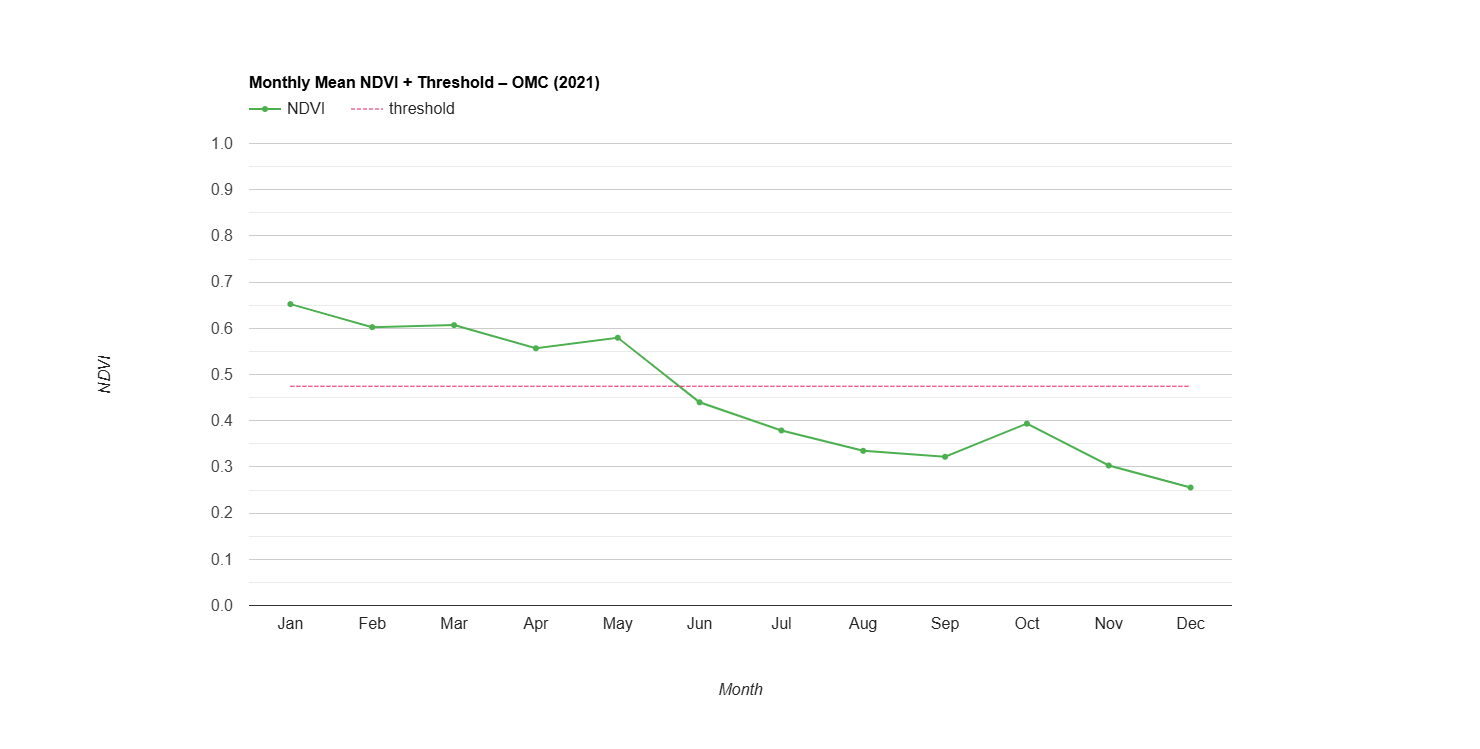

Supplement: Supplementary file 1 — Table S1: GPS coordinates and approximate sizes of livestock corrals (bomas) in Olare Motorogi Conservancy. Table S2: Grass species categorization and their ecological categories. Figure S1: Radial transect layout showing a 5 m buffer zone from the edge of the corral, with sampling in the Close (5–55 m) and Far (150–200 m) zones. Transects were established along three directions, each consisting of a Two‐50 m × 2 m belt transect, with a 5 m buffer zone maintained in each corral. Figure S2: Monthly mean Normalized Difference Vegetation Index (NDVI) for the OMC in 2021, with a 0.48 threshold line (dashed red line) to distinguish wet seasons (NDVI > 0.48) from dry‐season conditions (NDVI < 0.48). [file ECE3-16-e73855-s001.docx]
